# Essential Oils as Biofriendly Alternatives to Synthetic Insect Repellents

**DOI:** 10.3390/insects17060575

**Published:** 2026-05-31

**Authors:** Torben K. Heinbockel, Vonnie D. C. Shields

**Affiliations:** Biological Sciences Department, Towson University, Towson, MD 21252, USA; theinbo4@students.towson.edu

**Keywords:** essential oils, olfaction, repellent, arthropods, insects, behavior, green repellent, pesticides, pest, antimicrobial, therapeutic

## Abstract

Most plant-based essential oil repellent products currently available on the market utilize a “green” approach based on the volatile properties of essential oils. In general, these essential oils contain terpenes, terpenoids, phenylpropanoids or benzenoids that can be used to either (1) eliminate a human’s scent through a process called odor masking, or (2) interfere with an insect’s ability to detect a person’s scent through interaction with both olfactory receptors and odorant binding proteins. Additionally, many of the essential oil blends that have been developed have been shown to exhibit antimicrobial and therapeutic properties. The primary drawback to using essential oil-based repellents is that their protection times vary widely, and typically last only a short period of time due to the volatile nature of the active ingredients, as well as differences in concentration and formulation among products. Encapsulation, nano-delivery systems, and rationally designed blend combinations are being proposed as potential methods to delay the release of the essential oil active ingredients, thus extending the duration of effectiveness of the repellent product. Since essential oils represent complex mixtures, there is a possibility that resistance to the repellent active ingredients could develop differently than it would for single-active agents. However, before such resistance can be assessed, the repellents must undergo extensive safety evaluations, along with standardized efficacy assessments against Environmental Protection Agency (EPA)-approved repellent products, and ultimately, field trials must be conducted in areas where the repellents will be used to prevent vector-borne diseases. In addition to conducting these evaluations, the repellents must comply with existing state and federal pesticide regulations.

## Simple Summary

Plant-based repellents use essential oils made of terpenes, terpenoids, phenylpropanoids or benzenoids that either mask human scent or disrupt insect scent detection. Their environmental friendliness and frequent antimicrobial activity make them promising alternatives to synthetic repellents, but their short protection time remains a major limitation because the active compounds are highly volatile. Beyond pest control, many essential oils are also used in therapeutic contexts for effects such as relaxation, mood support, and symptom relief, which contributes to their broad consumer appeal. Current research aims to improve longevity through encapsulation and nanotechnology-based delivery systems. Because essential oils are complex mixtures, resistance may develop differently than with traditional single-ingredient repellents. Before these products become commercially viable, they must undergo extensive safety testing, standardized efficacy comparisons with EPA-approved repellents, large-scale field trials, and compliance with federal and state pesticide regulations.

## 1. Introduction

Insects are the most diverse group of organisms in terms of number of species. More insect species have been described in the literature in comparison to any other animal group. Insects belong to the Phylum Arthropoda. This phylum includes spiders, ticks, mites, scorpions, centipedes, crabs, and lobsters. Selected arthropod species benefit humans as a source of food or as pollinators, some of which provide honey. However, many arthropod species are considered dangerous and harmful to human health as they act as vectors for some of the most devastating human diseases. Vector-borne diseases transmitted by mosquitoes, ticks, fleas and other arthropods remain a major global health concern [1,2]. According to the World Health Organization [3], malaria was responsible for causing nearly 600,000 deaths in 2023. Likewise, other diseases such as dengue fever, Chikungunya, Zika and other viruses continue to cause large outbreaks. Humans have developed various methods to deter or destroy harmful arthropods capable of transmitting these diseases. These range from insect traps to poisons. Developing effective and safe repellents against arthropods is particularly important to prevent arthropod-borne diseases and is critical for human health and safety [4]. Since vaccines and therapeutic strategies are limited in their effectiveness, public health agencies emphasize prevention. The use of insect repellents is therefore a key component of personal protection. The most widely used synthetic repellent is DEET (chemical name, N,N-diethyl-meta-toluamide) developed by the U.S. military in the mid-20th century. It has been successful against pests, such as mosquitoes and ticks which cause diseases such as malaria and Lyme disease [5,6]. Insects, such as crickets, cockroaches, and ants, have omnivorous feeding preferences and can contaminate and destroy stored food products like sugar, flour, and grains with their feces [7]. DEET has raised environmental and human health risk concerns, which have prompted a search for more healthy alternatives [8].

More recently, some aromatic, plant-based essential oils are becoming more popular as alternatives to synthetic repellents against some arthropods [6,9,10,11,12]. Essential oils are volatile, aromatic compounds that can be extracted from plants by steam distillation or solvent extraction. They have a place in traditional medicine and have been used as fragrances, antiseptics, and remedies. Essential oils degrade rapidly in the environment and often have pleasant scents. They can be considered “green” or environmentally friendly repellents and have the potential to substitute harmful synthetic repellents. 

Essential oils work through the sense of smell of insects. Many insects have elaborate olfactory sense organs in the form of antennae [13]. Insect behavior is in large part regulated by odor molecules in the air that guide insects to find food, mates, hosts, or egg-laying sites. The cuticular insect antenna are located on the head of the animal and can be several inches long. These antennae bear sensory organs known as sensilla [13]. Individual sensilla have several receptor cells that can detect odors, taste molecules, temperature, or moisture. Insect repellents typically activate or inhibit odor-detecting receptor cells in these antennal sensilla. In mosquitoes, DEET acts by binding to the olfactory receptors. It disrupts host detection and prevents mosquitoes from biting a human host.

Concerns about DEET, as well as an increase in consumer preference for natural products, have stimulated interest in plant-based essential oils as green, biofriendly alternatives to conventional synthetic repellents. Given the negative side effects of synthetic insect repellents like DEET, it is important to determine if insect behavior can be regulated by specific biofriendly chemical compounds, like essential oils, that can potentially serve as insect repellents. Like DEET, many essential oils have been found to repel insects, as well as disrupt their olfactory-guided locomotory behavior [6,10,14]. Essential oils are complex mixtures of volatile organic compounds from plants. They can act as effective “green” biofriendly repellents by interfering with insect olfactory-guided behavior. The presence of monoterpenoids, sesquiterpenes, and alcohols, among other compounds present in essential oils, can be attributed to their repellent properties [15,16]. In addition, they can offer additional antimicrobial and therapeutic benefits and reduced ecological impact compared with conventional repellents and are effective against a variety of insect species.

## 2. Materials and Methods

This study used a literature-based research methodology by drawing on primary peer-reviewed articles, government guidelines, and authoritative reviews. Databases such as PubMed, the National Library of Medicine (PMC PubMed Central), Google Scholar, and the CDC Yellow Book were searched for publications on essential oils and insect repellents published up to 2026. Search terms included essential oil, repellent efficacy, olfactory receptors, nanoemulsion, citronella, catnip, neem oil, therapeutic, antimicrobial, and DEET environmental impact. Studies were selected that reported experimental results on the repellency of specific essential oils, described the molecular mechanisms of insect olfaction, or evaluated environmental or human safety of repellents. Secondary sources such as review articles and regulatory documents were also used to provide a broader context. Data from these sources were used to develop a structured argument addressing the research question.

## 3. Results

### 3.1. Synthetic Repellents

Synthetic insect deterrents, such as DEET, have been considered safe and the most effective insect repellents. It is effective against many different insect species and does not show selectivity as tends to be the case with essential oils. According to the U.S. Environmental Protection Agency (EPA), DEET has been deemed safe for human use [17] and recommended for use to repel biting pests such as mosquitoes and ticks. DEET has been found to protect people from mosquito-borne illnesses such as malaria, West Nile Virus, Zika virus, as well as tick-borne illnesses such as Lyme disease and Rocky Mountain spotted fever. DEET was developed by the U.S. Army in 1946, and it had a proven track record of reliability. It was made publicly available in 1957. In a report issued in 2014, the EPA maintained that DEET does not pose any risks to human health, non-target species, or the environment [18]. While the EPA does not issue any warnings against the use of DEET, other sources have reported concerns about its use. New reports demonstrate that DEET and DEET-like repellents are potentially harmful to human health and the environment [8]. DEET is not recommended for pregnant women or children younger than 6 months of age [11]. DEET persists in the soil and water and has been detected in groundwater and surface waters worldwide. Because DEET is long-lived in the environment, it is often detected in human urine, plasma samples, and drinking water [19,20,21,22]. It can enter the human body through the skin, where it is absorbed, or through inhalation of sprays. Since DEET is used so widely, its safety is a matter of public debate [8]. DEET can cause skin irritations, like rashes and itching, especially with prolonged use or when used in high concentrations. In certain individuals, exposure to DEET may trigger allergic reactions, potentially resulting in hives, swelling, or respiratory difficulties. Neurological complications linked to DEET exposure can include seizures and tremors and have been reported on rare occasions. Such incidents are exceedingly uncommon and are generally associated with misuse or accidental ingestion [23].

Although DEET is not regarded as a major environmental contaminant, high concentrations can negatively affect certain aquatic insects, fish, and plants. It is moderately toxic to aquatic organisms and may accumulate through food webs. In animal models and humans, exposure to DEET and its breakdown products have been found to cause toxic effects on the heart, immune system, and nervous system, as well as impaired function possibly because of oxidative stress [24,25,26,27]. In zebrafish embryos, exposure to DEET led to inhibited development. Another study found a relationship between DEET exposure and obesity-related outcomes in adult populations using data from the National Health aRmnd Nutrition Examination Survey (NHANES) [28]. The authors of this study suggest that food and exercise alone cannot account for the global rise in obesity. They suggest that environmental contaminants, such as the use of DEET, may be correlated directly. Other studies have found that DEET can impair human kidney function leading to hyperuricemia [29,30]. In hyperuricemia, elevated levels of uric acid can be found in the blood, resulting in health problems, such gout, kidney stones, and cardiovascular disease. Environmental exposure to DEET and its breakdown products have also been associated with increased mortality risks in females and non-Hispanic Black individuals [31]. Another negative side effect of using conventional chemical insecticides, such as DEET, over long periods, is the development of biological resistance thereby rendering them ineffective [10]. In contrast, essential oils have a complex molecular structure which makes it difficult or impossible for insects to develop resistance to them. Chemical research has resulted in many effective compounds that people have used in their daily lives. DEET is an example of such a chemical that is widely used because of its effectiveness. Available regulatory summaries indicate that DEET is used at the kilotonne-per-year scale for personal protection; U.S. domestic use alone is estimated at ~2.3–3.2 kt/year (active ingredient), and Europe has been estimated at ~0.4 kt/year, implying a conservative global lower bound of ~2.7–3.6 kt/year [32]. Three repellent chemical substances, namely picaridin, IR3535, and PMD have been registered by the U.S. EPA agency for their repellency properties and are commonly regarded as “proven” alternatives to DEET. With respect to picaridin, a member of the piperidine family, a 20% concentration was shown to provide complete protection (i.e., no tick bites) for approximately 12.6–15.3 h when tested against ticks in a laboratory setting [33]. The World Health Organization (WHO) documented that IR3535 (Insect Repellent 3535 or ethyl butylacetylaminopropionate) can be applied directly to human skin and clothing while controlled laboratory testing indicated that the duration of protection by IR3535 compares favorably to that afforded by DEET in terms of protection from biting flies [34]. PMD (p-menthane-3,8-diol or para-menthane-3,8-diol) is associated with products containing “oil of lemon eucalyptus” type repellents. The EPA has approved PMD for use on both skin and clothing. Research indicates that due to its lower volatility compared to other essential oils, it provides longer protection times like those obtained with some synthetic repellents compared to those derived from unprocessed essential oils [34].

### 3.2. Essential Oils as Natural Repellents

Essential oils are concentrated hydrophobic liquids composed of volatile phytochemicals extracted from leaves, flowers, bark, seeds, peels, or roots, most commonly via steam distillation. The compounds can dissolve in solvents like ethanol, ethers, fatty acids and have a relatively low molecular weight. Terpenoids, phenylpropanoids and as derivatives of short-chain aliphatic hydrocarbons could be classified to this group [35]. Essential oils contain mainly allylic mono-, bi-, or tricyclic mono- and sesquiterpenoids of different chemical classes including hydrocarbons, ketones, alcohols, oxides, aldehydes, phenols, or esters [36].

At room temperature, these oils are typically liquid, volatile, colorless, and usually less dense than water. In plants, they contribute characteristic fragrances that are emitted from the plants. They are widely used in the perfume and cosmetic industry, as well as in aromatherapy. Essential oils act as chemical signals to either control or modify the environment surrounding the plant. Functionally speaking, they can attract pollinators, repel predators, prevent seeds from germinating, or communicate with other plants by releasing signals chemically to, e.g., indicate the presence of herbivores [37].

Essential oils are effective and natural repellents against various insects and are not harmful to humans [38]. They have been found to be more effective than synthetic repellents like DEET. In this way, essential oils are superior to regular (synthetic) insect repellents, which are effective against insects but are negative to organisms and the environment [39]. Numerous essential oils, such as lemongrass, citronella, palmarosa, eucalyptus, lemon eucalyptus, and basil obtained from *Cymbopogon* spp., *Eucalyptus* spp., and *Ocimum* spp. (e.g.,) have been studied widely can weakly to robustly deter a variety of insects and do so by targeting the insects’ sense of smell, such as [6,40,41,42]. In many insect species, behavior is regulated by olfactory (smell) cues to locate food, mates, egg laying sites, and hosts. As an example, mosquitoes find humans by detecting carbon dioxide and human body odors with their antennae [43,44]. Volatiles of essential oils can confuse or overpower the odor receptors in the mosquito antennae, thereby preventing the insect from locating a person [45,46].

**Figure 1:**
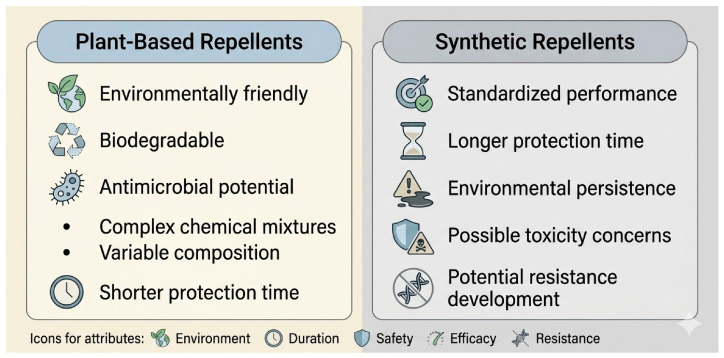
Some features of plant-based versus synthetic repellents.

**Figure 2:**
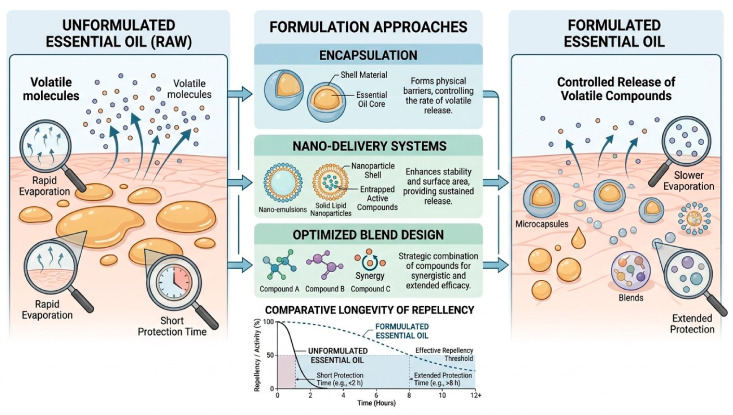
Formulation techniques to control the rate of evaporation of essential oils.

Essential oils can offer broad-spectrum protection against insects, as well as non-insect species, while others target specific pest species. As an added benefit, essential oils provide antibacterial, antifungal, insecticidal, and deterrent properties (Figure 1) [47]. They are becoming increasingly and widely accepted as promising “green” alternatives to synthetic chemicals, such as DEET. Essential oils are biodegradable and contain naturally occurring compounds (e.g., terpenes, terpenoids), however they degrade more quickly and do not persist as long in the environment as their synthetic repellent counterparts. They can be safer options for humans since they are less likely to cause adverse reactions than synthetic repellents for those with sensitive skin. However, essential oils may become toxic to both humans and pets if they are ingested or applied topically at high concentrations (e.g., eucalyptus, peppermint). Diluting essential oils may reduce their level of potency but can help to overcome concerns of toxicity. Likewise, applying essential oils to clothes instead of skin can provide a safe application method. Some essential oils, such as citronella, offer only a few hours of protection and must be reapplied frequently. Certain common essential oil-containing repellents, like citronella, require several applications per day because the oils evaporate relatively quickly. Further, there is the likelihood that the potency of essential oils may vary based on their concentration. This is not the case with the synthetic repellent DEET. In controlled arm-in-cage tests, ~20–24% DEET repellent provided about 4–5 h of complete protection prior to reapplication [5]. Advanced encapsulation and nano-formulation techniques can help oil-soluble substances, such as essential oils, to be more stable, have higher longevity, and to be steadily released through gradual evaporation. This can allow them to achieve longer repellant activity compared to synthetic options [10,11,22]. Moreover, using essential oils in blended formulations can help to enhance effectiveness by combining oils with varying complementary strengths, allowing broader, extended protection free from synthetic chemicals. Therefore, these innovations address concerns about extending the duration of effectiveness of essential oils (Figure 2).

**Figure 3:**
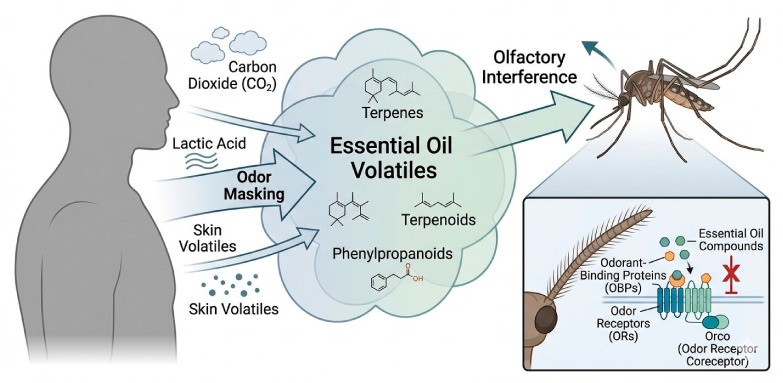
Mechanisms of essential oil repellency against mosquitoes.

Essential oils are volatile chemicals that interfere with the sensory receptors of insects [39]. Essential oils can interfere with insects’ olfactory sense through several mechanisms [39,42,48]. The volatile molecules released by essential oils can saturate the air around a host and, thereby, effectively prevent the host’s scent from being detected. The essential oils mask the smell of humans and make it harder for insects to detect their hosts [42]. The volatile components of certain essential oils, such as citronellal, linalool, eugenol, limonene, terpineol, and benzyl benzoate may be capable of binding to mosquito odorant-binding proteins (OBPs) (Figure 3). The OBPs may influence olfaction by either preventing the odor molecules from reaching their corresponding receptors and/or by modifying the function of these receptors to decrease their sensitivity to attractant odors. This can lead to a reduction in a mosquito’s ability to locate and feed on humans. Interestingly, many other important human attractants such as lactic acid and CO_2_ are perceived by mosquitoes through different pathways. Lactic acid is detected primarily through IR8a-dependent ionotropic receptors and CO_2_ is detected primarily through gustatory receptors. Therefore, it is likely that the volatile compounds of essential oils act by “blocking” or “desensitizing” odor receptors of mosquitoes resulting in their inability to locate their hosts [42].

Insect systems detecting odorants use odorant receptors (ORx) in conjunction with a co-receptor (Orco) that is both a receptor and an ion-channel within the complex. Upon binding of an odorant to an ORx, the ion channel in Orco opens resulting in activation of the sensory neuron responsible for the perception of the odor. As such, either allosteric agonists or antagonists that bind to either the ORx or the Orco could potentially be used as repellents to prevent the sensing of odors by insects. Additionally, repellents such as DEET, IR3535, and picaridin have been proposed to directly interact with specific mosquito odorant receptors (ORx), acting as agonists or antagonists that alter receptor signaling either on their own or by changing the receptor’s response to odorants like indole and 1-octen-3-ol [49]. Both compounds are of interest as they serve as odor cues which can reliably activate certain mosquito olfactory receptors [49]. Therefore, they offer two advantages; they represent actual stimuli with which mosquitoes respond to in the field, and they serve as readily available “reference ligands” in studies testing if a repellent modulates the response of olfactory receptors [49]. Indole and its analog, skatole, are produced through microbial activity or from decomposing organic matter and serves as a behavioral cue for mosquitoes, particularly when female mosquitoes choose egg-laying sites [49,50].

Many different essential oils have been shown to act as insect repellents such as citronella, lemongrass, eucalyptus, peppermint, clove, lavender, neem, geranium, thyme, and tea tree [39,51]. Even though the essential oils come from different plant families such as grasses, herbs, trees, and spices, they all share common chemical features, namely, they contain volatile compounds that insects find noxious or disorienting [52,53]. Each oil is composed of various secondary metabolite compounds that are of low molecular weight, have high volatility and are primarily lipophilic in nature [53]. More specifically, the major components of most oils are terpenes and oxygenated terpenoid compounds (specifically mono- and sesquiterpene types), with some oils containing additional phenylpropanoids such as eugenol [39,53]. The rapid evaporation of these molecules creates an intense olfactory plume near the surface of skin and clothing, and several of the molecules directly interfere with the normal functioning of insect sensory systems, creating confusing, aversive, or repellent signals that impede normal host-seeking and orienting behaviors [39,52].

Essential oils such as citronella, tea tree, geraniol, lavender, limonene, cinnamaldehyde, lemongrass, eucalyptus, and peppermint have been found to have strong repellent properties against mosquitoes and other insect species. Interestingly, citronella oil was officially registered as an insect repellent in the United States in 1948 by the EPA [54]. In comparison, while DEET was developed by the U.S. Army in 1946, it became available to the public only in 1957 [55]. Therefore, citronella oil was more widely used and registered as a repellent before DEET. Specific essential oils can deter specific insects and other pests. For example, citronella helps to keep mosquitoes away from humans [56], whereas eucalyptus oil is effective against ticks [57], and tea tree oil repels ants [58,59].

### 3.3. Chemical Classes of Essential Oils

#### 3.3.1. Terpenes and Terpenoids

Terpenes comprise a major class of natural products synthesized from C5 isoprene units and are generated in plants [60,61]. They are a class of natural organic compounds found in plants [61]. Terpenes occur at varying sizes, including C10 monoterpenes, C15 sesquiterpenes, and C20 diterpenes, with smaller terpenes generally exhibiting higher volatility and therefore contributing strongly to aroma and headspace chemistry [62]. In the form of essential oils, terpene hydrocarbons (such as limonene and pinene) are generally very volatile and contribute to a bright “top note” aromatic quality of the oil [62,63]. In ecological contexts, terpene emissions function as mediators of plant interactions by deterring herbivores, attracting mutualists such as pollinators, and participating in indirect defense signaling [60,61]. Many terpene constituents are chiral, and enantiomeric composition can influence both sensory perception and biological responses, including those of insect chemosensory systems [64,65]). Finally, terpene profiles are not static: unsaturated terpenes can undergo oxidation during handling and storage, leading to compositional drift that can alter odor and bioactivity and, in some cases, increase irritation potential, underscoring the need for controlled storage and reporting of sample history in experimental studies [47,62,63].

Terpenoids are very similar to terpenes. They have the same skeleton and contain one or more oxygen containing functional groups [62,66]. The presence of oxygen containing functional groups is important for discussion of repellents due to their effects on the physical properties of the compound such as its ability to evaporate and interact with receptors [62]. Alcohols, aldehydes, ketones, esters, oxides and ethers are all typical forms that terpenoids take [62]. The most common modification of terpenes occurs through oxidation which creates functional groups (alcohol, aldehyde, ketone, oxide, ester, ether) [35,62]. Citronellol, geraniol, linalool, and menthol are examples of alcohol terpenoids while citronellal and citral (a combination of neral and geranial) are prominent examples of aldehydes [62,66]. Menthone, camphor, and thujone are typical ketones while 1,8-cineole is can oxide or cyclic ether [62,66]. Examples of terpenoid esters include linalyl acetate, geranyl acetate, and menthyl acetate [62,66]. Oxidation of terpenes increases their polarity and decreases their volatility compared to their parent hydrocarbon compound [60,64]. This generally results in changes to the olfactory characteristics and longevity of an oil [66]. Variability in essential oil composition exists due to differences in plant genetics (chemotype), environmental conditions, harvesting methods, and extraction techniques [67,68]. Additionally, essential oil composition may be altered because of processing due to exposure to heat and/or time, allowing for conversion to other terpene pathways [62,66]. As many terpene components are susceptible to oxidation during storage, they will produce various products that can alter the aroma and possibly increase the irritation potential associated with use [35]. Thus, the importance of controlling the storage conditions of essential oils cannot be overstated to maintain consistency in the chemical structure of the compounds present [35]. In addition, for clinical use, essential oils must be standardized by setting and verifying specific percentage ranges for their “dominant constituents” so each batch has consistent composition, efficacy, and safety [69].

##### Monoterpenes and Monoterpenoids

**Figure 4:**
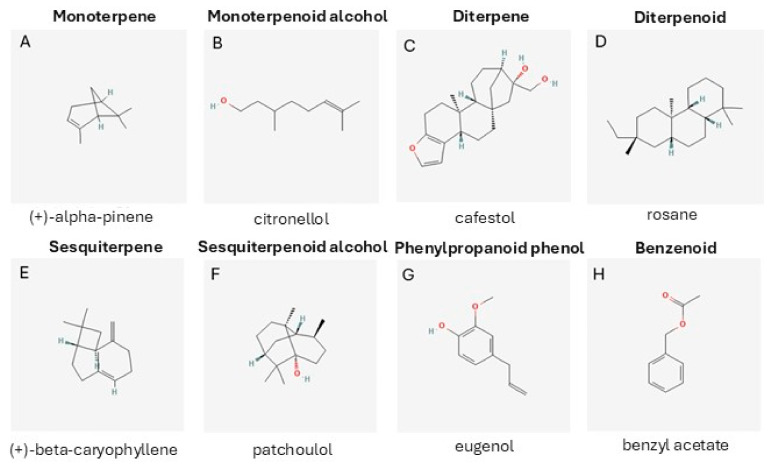
Examples of various chemical classes of essential oils and examples. (**A**), monoterpene (+ -alpha-pinene); (**B**), monoterpenoid alcohol (citronellol), (**C**), diterpene, (cafestol), (**D**). diterpenoid (rosane), (**E**), sesquiterpene (+ -beta-caryophyllene); (**F**), sesquiterpenoid alcohol, (patchoulol); (**G**), phenylpropanoid phenol (eugenol); (**H**), benzenoid, (benzyl acetate). Figures obtained from PubChem [82].

Monoterpenes are C10 terpenes that are created by combining two isoprene units (Figure 4A) [70,71] and can be found in abundance and high volatility in a wide range of essential oils [53], therefore monoterpenes typically compose the majority of what we experience when smelling an essential oil for the first time (“initial fragrance” or “first impression”) [70]. Monoterpenic hydrocarbon molecules, such as limonene, alpha pinene, and myrcene) tend to have low polarity and will therefore evaporate at a relatively rapid rate and contribute the strong citrus, pine, and resinous-green aromatic character to their respective essential oils [53,72]. Representative monoterpene hydrocarbons include limonene and the pinene isomers, along with compounds such as α-pinene, β-pinene, myrcene, γ-terpinene, p-cymene, and sabinene [53]. In many oils, these constituents contribute strongly to the initial odor impact and rapid evaporation behavior that can shape short-term repellent performance [72,73].

Monoterpenoids are oxygen-containing ten-carbon molecules made from two isoprene units (Figure 4B) and represent a large portion of many essential oils, and several classic plant-based repellents fall into this category [47,74]. Monoterpenoids are oxygen-containing ten-carbon molecules made from two isoprene units and represent a large portion of many essential oils. They have changed to include alcohol, aldehyde, ketone, and oxide functions. A few representative monoterpenoid types include linalool (alcohol), citronellal (aldehyde), menthol (alcohol), camphor (ketone), and 1,8 cineole (oxide) and many plant-based repellents fall into this category [47,74]. For example, geraniol and linalool have shown to be spatial repellents in laboratory tests; for example, in an indoor/outdoor diffusion chamber experiment, linalool was found to reduce mosquito trap captures more than citronella, while geraniol had the highest capture reductions of the three [75]. Para-menthane-3,8-diols (PMD), which is found in oil of lemon eucalyptus, has been described as highly effective and has a longer duration of action compared to other repellents [73,76]. Repellent effects of essential oils are generally due to mixtures of monoterpenoids (for example citronella type); however, the length of protection varies significantly across studies, and when evaporation is slowed through formulation, protection times may be longer [73].

Catnip’s nepetalactones are some of the most researched natural insect repellents which include iridoid monoterpenoids (atypical monoterpenoids). Insect repellents from catnip specifically stimulate the irritant TRPA1 receptors; these stimulated responses result in a powerful avoidance response and explain why catnip repellents will repel such a wide array of insects [77]. Products containing catnip with high levels of nepetalactone or isomers of nepetalactone have been demonstrated through standardized bioassays to repel mosquitoes, ticks and mites; in addition, they often compare favorably to commonly used insect repellents [78,79,80].

Oxygenation of monoterpenes increases their solubility in water (polarity) and decreases their volatility when compared to their parent monoterpenes [81]. As such, monoterpenoids tend to give off less vapor than their hydrocarbon counterparts. In general, they will also tend to smell more pleasant, as well as smell less fleeting and be more persistent on skin and other surfaces. Since monoterpenes and monoterpenoids are both volatile and chemically active, their relative concentrations may vary depending upon plant chemotype, processing techniques (i.e., harvest and distillation methods), and storage conditions (oxidative aging) [35,67]. Changes in these factors can influence both the scent characteristics of essential oils and their effectiveness for the desired purpose [67].

##### Diterpenes and Diterpenoids

Diterpenes are terpene compounds made up of C20 terpene hydrocarbons formed by four isoprene units (C5H8) (Figure 4C). Diterpenes chemically consist only of carbon and hydrogen. They are usually non-polar, commonly found as resins, and are much less volatile than monoterpenes and sesquiterpenes [83]. The large molecular weight and lower volatility make them relatively rare in the lighter, more volatile fractions of steam distilled plant extracts, and more commonly found in the resins [83].

Diterpenoids are oxygenated (functionally modified) versions of diterpenes. They retain the C20 terpene backbone of the parent diterpene but contain one or more oxygen- containing functional groups, such as an alcohols (-OH), ketones (C=O), aldehydes (-CHO), carboxylic acids (-COOH), esters (-COO-), or ether/epoxides (R-O-R) (Figure 4D) [62]. Addition of oxygen to a diterpene structure usually results in increased polarity of the compound, and changes in how the compound functions, such as increasing solubility, increasing reactivity, and providing for possible hydrogen bond interactions with biological targets [53,81]. Like diterpenes, diterpenoids may also be found in the less-volatile portions of plants; however, there may be trace amounts present in essential oil based on the specific plant species and extraction methods used [62,83]. They act primarily in a feeding-contact/deterrent manner, but not as a primary volatilized repellent [84,85].

The clerodane diterpenoids are an example of diterpenoids which have been extensively studied, showing both feeding deterrent and repellent activity toward insects using various assay methods, and showing structural activity relationships (SARs) based upon the presence of specific oxygenated functional groups (e.g., certain types of furan diterpenes and lactones) [84,86,87]. An extensive review in Natural Product Reports notes the wealth of research into this area, with over 200 natural clerodane diterpenes, as well as many semi-synthetic derivatives, having been tested for their insect feeding inhibitory (and related plant protective) activities [85]. While clerodanes represent the most well-investigated diterpenoid family in terms of insect feeding inhibition and related plant protection, other diterpenoid families, e.g., abietanes, e.g., ferruginol-related oxides isolated from *Taxodium distichum*, have been reported to inhibit termite feeding (in particular, subterranean termites) and thus exhibit significant “stay-away” repellency [88,89]. Furthermore, botanical insecticides (e.g., rosemary) containing phenolic diterpenes (e.g., carnosic acid and carnosol), illustrate how diterpenoids may be employed in long lasting defense mechanisms of plants against insects, and serve as an example of how diterpenoids may provide defense mechanisms to protect plants in commercial formulations [90].

##### Sesquiterpenes and Sesquiterpenoids

Sesquiterpenes are classified as C15 terpenes and are formed from a combination of three isoprene units (Figure 4E) and primarily synthesized in plant cells via the intermediate product farnesyl pyrophosphate (FPP) [60]. The sesquiterpenes found in essential oils tend to have lower volatility than monoterpenes and frequently provide the woody, spicy, resinous and earthy aromas that remain after the more volatile compounds in the oil have evaporated [91,92]. As such, the structural diversity of sesquiterpenes is tremendous, due to the various possible cyclization pathways for FPP [60]. Therefore, they may exist in either acyclic forms (e.g., farnesene), monocyclic structures, or complex bicyclic/tricyclic ring systems (e.g., β-caryophyllene, humulene, and bisabolene). Due to their hydrocarbon composition, sesquiterpenes are generally non-polar and may act as “fixatives” in blends to slow the rate of total loss of aroma profiles [91,92], but they also undergo oxidation reactions over time which contributes to why the aromatic characteristics and irritation potential of some essential oils may change over time when stored [35,93,94].

Sesquiterpenoids (oxygenated C15 terpenes made from three isoprene units) (Figure 4F) show up in many essential oils and are interesting as repellents because they are often less volatile than monoterpenes, which can translate to longer-lasting “bass note”, “stay away” signals on skin or surfaces [81]. Several specific sesquiterpenoids have strong evidence: nootkatone (a grapefruit/Alaskan yellow cedar-derived sesquiterpenoid ketone) repelled *Aedes aegypti* in human-arm biting assays, and work around it has supported its development into registered repellent/insecticide uses in the United States [38,95]. Patchouli alcohol (a sesquiterpenoid alcohol from *Pogostemon cablin*) has also been reported to provide high levels of protection against major mosquito vectors at tested doses and exposure times [96]. Other sesquiterpenes with similar long-lasting behavioral effects are β-caryophyllene, humulene, and germacrene [97,98]. Beyond mosquitoes, sesquiterpenoids and related sesquiterpenes can function as semiochemicals that drive avoidance behavior in other pests: for example, (E)-β-farnesene is a classic aphid alarm signal that triggers dispersal and escape, which is essentially “repellency” at the behavioral level [99]. Plant-emitted sesquiterpenes like (E)-β-caryophyllene are part of defensive blends that help plants reduce herbivore pressure sometimes indirectly by recruiting natural enemies, illustrating how this chemical class is deeply tied to “keep pests off” ecology [100].

#### 3.3.2. Phenylpropanoids

Phenylpropanoids are aromatic, non-isoprenoid-derived, and particularly prevalent in oils associated with spices and typically consist of either phenolic or aldehyde structures (Figure 4G). They contain a six-carbon atom and three-carbon atom skeleton often derived from the phenylalanine biosynthetic pathway. They are often cited as being active in bioassays involving the behavior of insects. They include many of the most recognized “spice oil” molecules found within essential oils, such as eugenol (clove), (E)-cinnamaldehyde (cinnamon), and (E)-anethole/estragole (anise and fennel) [47,62,101,102].

A repeated finding among studies focused on repellents is that many of the phenylpropanoids have the potential to elicit strong avoidance and inhibit biting behaviors at biologically relevant concentrations, and sometimes alongside causing toxicity, making these compounds of interest as “dual action” botanicals [73,103]. There is good documentation for the repellent activity of eugenol against the tick species *Ixodes ricinus* [104]. A subsequent Structure Activity Relationship (SAR) study initiated with eugenol as a starting compound identified many structurally similar phenylpropanoid compounds which demonstrated repellent activity in a bioassay using the same tick species [104]. Some of the repellent phenylpropanoids were tested at concentrations that were equivalent to (and in some cases lower than) the lowest concentration tested for DEET in this test. Eugenol was used again in a separate tick study, along with methyl eugenol, another phenylpropanoid, to assess repellent activity and the effectiveness of the repellent activity when applied at concentrations of 1–2% [105]. The authors report that both eugenol and methyl eugenol produced significant repellent activity at 1–2%, and that their activities were comparable to 7% DEET. Cinnamaldehyde has strong repellent activity in *Haemaphysalis longicornis* ticks. Furthermore, cinnamaldehyde alters the electrophysiological responses of the Haller’s organ in *H. longicornis*, and the repellent activity of cinnamaldehyde is dependent upon the presence of a specific ionotropic receptor, referred to as HL-IR, since knocking out HL-IR results in a reduced repellent activity and sensory response [106]. Finally, phenylpropanoids also demonstrate repellent activity against insects that can damage stored food. Specifically, in four-way olfactometer assays with the confused flour beetle, *Tribolium confusum*, anethole produces repellent activity at low concentrations, and the essential oil containing anethole will suppress attraction to the beetle aggregation pheromone [107].

#### 3.3.3. Benzenoids

Benzenoids are plant volatile compounds with a benzene ring structure, including benzoates, salicylates, and anthranilates (Figure 4H) [108,109]. They act as “stay away” signals (“keep-away” or repellent cues). They function most commonly as spatial repellents or oviposition-deterrents and rarely as contact-toxins [110]. Methyl benzoate, a floral benzenoid is an example of this type of chemical that was tested as a spatial repellent/oviposition deterrent for pests such as spotted wing *Drosophila* and specifically framed as a repellent in “push-pull” tracking and field style approaches to pest management [111]. Methyl salicylate, another benzenoid, reportedly acts to repel gravid insects and/or provides information to plants regarding the presence of an insect and contributes to oviposition deterrence signaling [110]. Additionally, benzenoids have applications beyond the realm of insects. For example, methyl anthranilate is identified within the applied literature as a strong avian chemosensory (trigeminal) irritant and has been studied as an avian deterrent in several studies [112]. Benzyl benzoate, (a general term for aromatic esters) is noted by many researchers to possess acaricidal activity and has also been referenced as a tick/chigger/mosquito repellent in pharmacology/toxicology resources [113,114].

### 3.4. Chemical Composition and Dominant Constituents of Essential Oils as Repellents

Even though each essential oil may contain dozens or even hundreds of trace minor constituents [53,62], repellency tends to cluster around a small number of recognizable chemical profiles. In most oils, the profile is driven by only a few dominant compounds that account for most of the total GC–MS peak area. Because these major constituents largely determine the oil’s volatility and odor, they also shape the core “baseline repellent signature” of the blend [62]. The main chemical profiles commonly implicated in repellency are oxygenated monoterpenoids (e.g., aldehydes, alcohols, oxides/ethers, and ketones) and aromatic phenylpropanoids, with some oils also containing lactone-type constituents depending on botanical sources and processing [47,62,115]. Although dozens to hundreds of minor constituents may be present, the dominant peaks in an oil’s chromatogram reflect most of the oil’s volatility and odor signature and can account for a sizeable portion of behavioral bioactivity observed in assays [53,116]. Sometimes minor consitutents (present <1%) can strongly influence the “odor character”and act as synergists and enhance pentration into the antenna, bind to OPBs, or act downstream at receptors [117]. An oil’s composition is not static and can vary with genetics (chemotype), geographic location, harvest timing, and methods/conditions of distillation or expression [67,68,118]. As a result, more recent repellency discussions increasingly pair bioassay outcomes with GC–MS chemical profiles, since compositional differences within “the same oil” can plausibly shift repellency rankings across studies [116,118].

Citral-based, and citronellal-based essential oils, are two types of essential oils that are primarily composed of monoterpenoid aldehydes [47,62]. Citral comprises neral and geranial [62], while citronellal is a direct aldehyde form of citronellol [119,120]. Oils of this type are typically very potent and have strong odor mediated effects [73]; however, they also tend to evaporate rapidly and therefore have limited duration when used alone. Thus, the longevity of an oil that has evaporated quickly would need to be extended through either formulation or application strategies that slow evaporation and prolong release [73]. On the other hand, many repellent studies include oils that contain monoterpenoid alcohols such as geraniol, citronellol, linalool, and menthol [47,62]. This is because all the above-mentioned compounds are found alcohols such as geraniol, citronellol, linalool, naturally in a variety of plants and are volatile and possess significant sensory properties that contribute to the measured repellency [47,75].

A second common motif for repellents is the presence of phenolic monoterpenoids, specifically thymol and carvacrol, which are typically found in oils derived from plants in the Lamiaceae family (e.g., thyme and oregano), as well as in some of their respective chemical profiles [121,122]. It is common to find reports of high bioactivity for thyme- and oregano-type oils in insect-related contexts, consistent with the broad bioactivity of these phenolic monoterpenoids and their rich essential-oil mixtures [73,123]. These oils also commonly contain abundant accompanying monoterpene hydrocarbons such as p-cymene and γ-terpinene, which are frequently present alongside thymol/carvacrol and can vary substantially with chemotype [122,124]. In addition, it is common to find phenylpropanoid-rich oils being tested, particularly eugenol-dominant clove-type oils and cinnamaldehyde-dominant cinnamon-type oils, because a single compound can constitute a large fraction of the total chromatographic area and thus strongly shape odor, volatility, and measured bioactivity [47,125,126].

### 3.5. Chemical Signatures of Repellent Essential Oils

**Figure 5:**
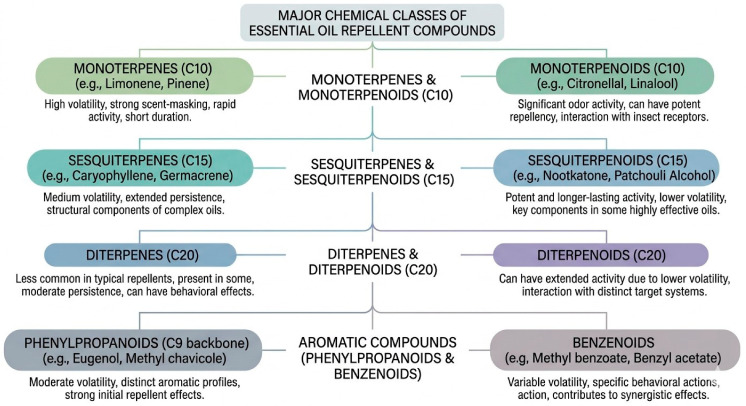
Classes of essential oils and their repellent properties.

Essential oils commonly referenced as repellents tend to group into specific chemical signatures: aldehyde-rich *Cymbopogon* oils (e.g., citronellal or citral); phenolic *Lamiaceae* oils (e.g., thymol or carvacrol); phenylpropanoid-rich spice oils (e.g., eugenol or cinnamaldehyde); lactone-dominant catnip oil (nepetalactones) [79] and a variety of oxygenated “medicinal” profiles dominated by cineole, camphor, or related compounds (e.g., eucalyptus, rosemary, and sage oils) (Figure 5). This chemical framework also provides a practical connection between composition and bioassay interpretation: rather than simply treating “oil identity” as a label, the dominant constituents can be utilized as a mechanistic basis to understand why oils within the same common-name category may exhibit differing levels of repellency across studies. Composition is not static. Essential oil profiles vary with plant genetics (chemotype) [122,124], environment, harvest timing, and extraction conditions, and can also shift during processing (heat and time) and storage. Many common terpene constituents oxidize during storage, and oxidation products can change odor and increase sensitization or irritation potential, which is why reporting storage conditions and sample history matters in experimental work.

While raw lemon eucalyptus essential oil (from *Corymbia citriodora*) has low PMD (p-menthane-3,8-diol) levels, oil of lemon eucalyptus is refined to specifically boost PMD levels. Repellent activity is attributed to PMD, an effective oxygenated monoterpenoid (diol) [9,76,127]. In most repellent applications, PMD is treated as the primary active because PMD-based products, derived from lemon eucalyptus sources, can contain it at high levels and it tends to persist longer than highly volatile monoterpene hydrocarbons. As a result, compositional descriptions and many practical evaluations in this category focus specifically on PMD as the dominant component rather than on the full essential oil bouquet [9,76,127]. From a chemical standpoint, the distinction is that the PMD-centered profile is oxygenated and therefore less “top-note volatile” than limonene- or pinene-dominated citrus and conifer oils, which evaporate very quickly and provide the bright first odor. This helps to explain why PMD-based repellents are commonly discussed separately from most unformulated essential oils [9,47,127].

Citronella oils (*Cymbopogon* spp.) represent one of the most frequently referenced essential oils in repellent assessments and are chemically characterized by a triad of monoterpenoid constituents: citronellal (an aldehyde), citronellol (an alcohol), and geraniol (an alcohol). Additionally, most citronella oils contain measurable amounts of geranyl acetate (an ester) and lesser amounts of monoterpene hydrocarbons that affect scent and volatility. Due to their strong odor and rapid evaporation, citronella oils are often used as the “classical” example of botanical repellents [128,129]. As a result, the majority of studies examining citronella reference that the speed of evaporation is a common problem, and thus studies referencing citronella frequently report that formulation techniques that control the rate of evaporation (gels, lotions, microencapsulation, or blends of fixatives) can be as important as the oil itself [130] (Figure 2).

Lemongrass oils (also *Cymbopogon* spp.) are commonly grouped with citronella oils in repellent assessments; however, the chemical characteristics of lemongrass and citronella oils are different in that lemongrass oils are citral-dominant [131]. Citral is not a single compound; rather it is a mixture of two monoterpenoid aldehydes, geranial and neral, and in many lemongrass oils, these two peaks are among the largest in the chromatogram [132]. Like citronella, lemongrass can also contain geraniol and geranyl acetate, along with minor amounts of monoterpene hydrocarbons [131]. Since citral is highly volatile and chemically reactive relative to some other terpenoids, discussions of lemongrass as a repellent frequently reference strong initial repellency activity, the potential for oxidation/reaction of the citral during storage, and the significance of the method of delivery to achieve meaningful protection durations [35].

Catnip oil (*Nepeta cataria*) is often referenced as a botanical repellent because the chemical identity of catnip oil is unusual in that it is dominated by isomers of nepetalactone, which are lactone-type monoterpenoid derivatives [79]. Frequently, the nepetalactones are the dominant components of the oil, with the remaining components of the oil consisting of smaller amounts of related terpenoids [78]. Catnip is a useful comparison to oils such as citronella and lemongrass, which are characterized by a broad spectrum of monoterpenoids, to demonstrate that strong repellency can be attributed to a single dominant scaffold (i.e., nepetalactone).

Clove oil (*Syzygium aromaticum*) represents a different biosynthetic class of oils than citronella and lemongrass oils, as clove oils are typically dominated by eugenol, a phenylpropanoid phenol [133]. Often, clove oils contain measurable amounts of eugenyl acetate (an ester) and β-caryophyllene (a sesquiterpene hydrocarbon), in addition to the dominant eugenol. Eugenol-dominant oils are commonly referenced in repellency screening studies because of their ease of identification using GC-MS and their potency. Additionally, clove oils serve as a reference point that not all potent repellent oils are primarily composed of terpenes [134]. Phenylpropanoids can dominate a repellent oil and exhibit significantly different volatility and persistence properties than monoterpene aldehydes or alcohols.

Cinnamon essential oils (*Cinnamomum* spp.) are frequently included in repellency screens and can reduce mosquito attraction or biting under laboratory bioassays [38,135]. However, it is important to distinguish bark oil from leaf oil, because their dominant constituents can differ substantially, meaning “cinnamon oil” may refer to chemically different materials depending on the plant part and source [27,136]. Cinnamon bark oils are commonly reported as (E)-cinnamaldehyde-dominant, with cinnamaldehyde appearing as the major aromatic aldehyde peak in GC–MS profiles [126,136]. In contrast, cinnamon leaf oil is often eugenol-rich, which makes some leaf oils chemically closer to clove-type profiles than to cinnamaldehyde-dominant bark oils [137]. This distinction matters when interpreting repellency results as “cinnamon oil” may be referring to a chemically distinct material depending on the source and method of extraction of the oil. Repellent activity associated with cinnamon-type oils can be strong in screening studies, but the likely active repellent constituents depend on whether the oil is primarily cinnamaldehyde-dominant or eugenol-dominant [38,116].

Thyme oil (*Thymus vulgaris*) and oregano oil (*Origanum* spp.) are frequently referenced as highly bioactive essential oils and are often grouped together as “phenolic Lamiaceae oils,” largely because many chemotypes are dominated by the phenolic monoterpenoids thymol and/or carvacrol [122,138,139]. Thyme is often represented as a thymol chemotype, where thymol is a dominant peak, commonly accompanied by substantial p-cymene and γ-terpinene [140,141,142]. These hydrocarbons are often discussed as biosynthetic precursors associated with phenolic thyme profiles, and they also strongly shape aroma [122]. Oregano is often represented as a carvacrol chemotype, where carvacrol is dominant and p-cymene and γ-terpinene are commonly present at appreciable levels [123,138,143]. These oils are frequently used to illustrate two points: (i) phenolic monoterpenoids can be highly active in insect-related assays, and (ii) chemotype matters, since both genera can also occur in non-phenolic chemotypes, including linalool-rich profiles, which may behave differently in repellency testing [142,144].

Peppermint oil (*Mentha × piperita*) is commonly evaluated as a repellent and is generally characterized as having a menthol (a monoterpenoid alcohol) and menthone (a monoterpenoid ketone) dominant profile, with additional contributions from isomenthone, menthyl acetate, and smaller amounts of monoterpene hydrocarbons [145]. Peppermint is often included in botanical repellent comparisons because it appears frequently in plant-based repellent discussions and has documented mosquito repellency in laboratory and human-skin style evaluations, while essential oils in general tend to show limited duration due to their volatility [73]. Interestingly, “mint oils” are not interchangeable. Spearmint (*Mentha spicata*) is carvone-dominant (often with limonene), and other *Mentha* species can alter the balance between alcohols, ketones and other monoterpenes. Therefore, it is important to specify the type of mint (or hybrid) and dominant constituents when comparing the repellent effectiveness of different oils.

Eucalyptus oils are frequently included in repellent studies, but their chemical composition is highly species- and chemotype-dependent [146,147]. Many eucalyptus oils (particularly cineole-dominant oils) are dominated by 1,8-cineole (eucalyptol), an oxygenated monoterpenoid oxide/ether, often with supporting monoterpene hydrocarbons including α-pinene and limonene [148,149]. Other eucalyptus species can be more citronellal-dominant, resulting in a profile that can be more similar to aldehyde-rich citronella than to cineole-dominant oils [147,150]. Eucalyptus is a good example of how the botanical name and dominant constituents of an oil are more important than the common name, as “eucalyptus oil” can refer to chemically distinct materials that may not have similar repellent activities.

Rosemary oil (*Salvia rosmarinus*, formerly *Rosmarinus officinalis*) is frequently referenced in insect behavior and repellent studies [73,151,152]. Rosemary oils are notable for exhibiting multiple chemotypes, with composition shifting across plant source, geography, and processing procedures. Many rosemary oils contain 1,8-cineole among the dominant peaks, often accompanied by α-pinene and oxygenated monoterpenes including camphor, and sometimes borneol or verbenone based on plant source and distillation [141]. This mixed cineole-plus-camphoraceous profile is often interpreted as potentially useful for repellency, but reported performance can vary substantially across studies, which reinforces the importance of chemotype, doe, and especially formulation or solvent system in determining protection time [73,151,152]. Rosemary is also commonly used to illustrate mixture effects in essential oils, since oils often contain multiple mid-to-high abundance constituents rather than a single dominant compound, and bioactivity may reflect the blend rather than any one component [141,152].

Sage oil (*Salvia officinalis*) is another Lamiaceae oil that is commonly evaluated in insect-related studies [153,154]. Sage oils are often characterized by dominance of oxygenated monoterpenoids, specifically thujone isomers (frequently α- and β-thujone), camphor, and 1,8-cineole in varying proportions [155,156]. Sage oil is a good example of why composition must be handled carefully: the thujone/camphor/cineole balance can shift substantially across samples (chemotype, harvest stage, geography, and processing), and those shifts can reasonably change bioactivity and measured repellency in behavioral assays [155,156]. When sage is referenced in repellency screening studies, its performance is often interpreted through the context of its oxygenated-ketone and oxide-dominant chemistry rather than through the context of monoterpene hydrocarbon-dominant chemistry [155].

Geranium oil (typically *Pelargonium graveolens*, “rose geranium”) is frequently included in botanical repellent testing, in part because it shares key odorant constituents with “citronella-type” profiles and has shown measurable repellency in both mosquito and tick assays [57,152,157]. Geranium oils are often rich in citronellol and geraniol (monoterpenoid alcohols), with esters such as citronellyl formate and other related citronellyl/geranyl esters varying by oil and source [158,159,160]. This places geranium within a broader “citronellol/geraniol alcohol” chemistry that is repeatedly associated with repellency in insect assays, including studies where geraniol itself performs strongly as a botanical repellent [75]. Differences in alcohol-to-aldehyde balance and ester content across oils can shift evaporation behavior and perceived persistence, which matters because the practical duration of protection for many essential oil repellents is often limited by volatility unless formulations slow release [73].

Lavender oil (*Lavandula angustifolia*) is frequently referenced in repellent studies, in part because it is widely used in personal-care and fragrance contexts and has a well-established chemical “fingerprint” in GC-MS analyses [73,161]. Many lavender oils are characterized by linalool (a monoterpenoid alcohol) and linalyl acetate (an ester), with smaller contributions from terpinen-4-ol, lavandulyl acetate, and minor monoterpene hydrocarbons that vary by cultivar and production conditions [161,162,163]. Lavender oil’s typical alcohol-plus-ester profile can provide measurable repellency in certain assays, but outcomes and protection times are variable across studies and often depend strongly on formulation, since evaporation can limit the practical duration of many essential-oil repellents [57,73,152].

### 3.6. Essential Oils as Antimicrobial and Therapeutic Agents

Many essential oils have antimicrobial features, in addition to serving as insect repellents. Essential oils can demonstrate antimicrobial activity as they contain active compounds, like terpenes, phenols, and aldehydes, which inhibit a broad range of microorganisms, including bacteria, fungi, and viruses. Among the ~250 commercially available essential oils, about a dozen possess high antimicrobial potential [164]. Examples include oils from thyme, oregano, tea tree, and clove that have been effective against pathogens like *Staphylococcus aureus*, *Escherichia coli*, and *Candida albicans* in laboratory settings [165]. Essential oils disrupt microbial cell membranes which causes leakage and cell death [166]. They also inhibit enzyme function and interfere with cell signaling. Essential oils can be used together with antibiotics to enhance their effect. They can be eco-friendly alternatives to synthetic antimicrobials, especially in food preservation, healthcare, and cosmetics. However, essential oils are not designed to be specifically antimicrobial compounds, they are likely to be inferior to any commercially available antimicrobial medication. Nevertheless, the antimicrobial properties of essential oils provide an added benefit that is not present with conventional synthetic insect repellents.

Essential oils can often have calming, healing, and mood-enhancing effects and are known to reduce stress and anxiety by regulating the limbic system, the part of the brain that influences emotions and memory [167]. For example, lavender, chamomile, and ylang-ylang are known for their calming and relaxing effects and studies have shown that inhaling essential oils, such as lavender oil, can significantly reduce anxiety and improve sleep quality [167,168,169]. In high-stress environments, essential oils serve as a natural solution for stress relief and provide mental health support [167]. As far as respiratory health is concerned, eucalyptus and peppermint are frequently used in aromatherapy for their benefits to respiratory health. They aid in clearing airways and reducing congestion [36,170]. Furthermore, peppermint oil can relieve tension headaches. This effect is likely due to its menthol content which cools the respiratory passages and leads to muscle relaxation [36,171]. Essential oils help with cognitive health, mental focus and concentration and have been used by aromatherapists to calm patients suffering from dementia, as well as to improve patients’ cognitive functions (mixtures of lemon and rosemary and lavender and orange essential oils) and neuropsychiatric symptoms (e.g., lemon balm and lavender essential oils) [167]. Rosemary and peppermint essential oils are routinely used to enhance alertness and cognitive performance [167,170]. Tea tree and frankincense have antibacterial, antifungal, and immune boosting properties [172], thereby providing immune support. While many studies on aromatherapy are not fully validated in terms of reliability [169,173], this may create a problem when essential oils are applied in the clinic where consistent results are required. Despite these concerns, however, essential oils are known for their function in aromatherapy, making them versatile options in therapeutic treatments.

## 4. Conclusions

Essential oils are becoming increasingly and widely accepted as promising “green” alternatives to synthetic chemicals, such as DEET, as synthetic repellents are considered detrimental to beneficial insects, wildlife and ecosystems and cause adverse effects on humans. There is evidence that several essential oils are effective in repelling a variety of insect and pest species, such as mosquitoes, fleas, and ticks. Essential oils are thought to interfere with antennal olfactory receptors, making it difficult for them to locate human hosts.

Some essential oils can offer broad-spectrum protection against insects, as well as non-insect species, while others target specific pest species. As an added benefit, essential oils provide antibacterial and antifungal properties and may provide relief and/or disinfection from insect bites or other skin irritations. Having said this, essential oils are highly concentrated solutions and must be used with caution. While pure essential oils may repel insects, they can induce skin irritation (e.g., tea tree, eucalyptus, clove). Skin irritation, allergic reactions, or toxicity have been reported when used improperly, especially in children or pets. Some essentials oils, such as oregano or cinnamon, when applied directly to the skin, without proper dilution, may be particularly harsh. However, proper dilution and blending of essential oils with carrier oils can help to reduce the potential for skin irritation or toxicity while maintaining their efficacy over time. Applying essential oils directly to clothing instead of skin can provide a safe alternative application method to avoid skin irritation due to direct topical application and reduce the chance of harmful exposure to humans. In addition, essential oils may become toxic or pose adverse reactions to both humans and pets if they are ingested or applied topically at high concentrations (e.g., eucalyptus, peppermint). While diluting essential oils may reduce their level of potency, health risk concerns regarding toxicity and reactions can be minimized.

Essential oils may be less effective or degrade more rapidly as compared with their synthetic repellent counterparts. Some essential oils, such as citronella, offer only a few hours of protection and must be reapplied frequently. The potency of essential oils may vary depending on their concentration. With new delivery systems being manufactured, the stability and longevity of essential oils have increased drastically, making them comparable to synthetic chemical counterparts.

Essential oils are thought to have therapeutic effects on physical, emotional, cognitive, and psychological well-being. Depending on the essential oil, aromatherapy can help reduce stress, anxiety, and depression, improve sleep quality, and enhance overall mood. These significant benefits outweigh the anecdotal reports of negative side effects of aromatherapy. Essential oils provide a multifaceted tool for overall well-being and are appreciated in holistic therapeutic health practices. While producing essential oils involves substantial amounts of plant material and may cause a strain on natural resources and potentially limit their availability, more widespread use of essential oils can boost the industry involved in producing the required plant material and manufacturing essential oils. This, in turn, can create a boost to the economy. While essential oils have been shown to promote emotional and physical health, there are valid concerns regarding their safety, even when they are used in aromatherapy. Scientific evidence for use of essential oils in aromatherapy is not as robust as that for synthetic aroma chemicals. Typically, the latter are formulated to imitate natural aromas found in plants and produced at minimal cost.

Taken together, essential oils are multifunctional natural products that can provide effective insect repellency alongside broad antimicrobial activity, with many showing additional therapeutic benefits in traditional and modern use. Their chemical diversity gives them a wide range of bioactivities, making them useful across contexts from personal protection to hygiene and complementary health applications. At the same time, their performance depends on composition and formulation, so standardized profiles and evidence-based dosing are key for reliable real-world use.

## Data Availability

The original contributions presented in this study are included in the article. Further inquiries can be directed to the corresponding author.

## Abbreviations

The following abbreviations are used in this manuscript:

EPA	Environmental Protection Agency
WHO	World Heath Organization
DEET	N,N-diethyl-meta-toluamide
FPP NHANES	Farnesyl Pyrophosphate National Health and Nutrition Examination Survey
DDT	Dichlorodiphenyltrichloroethane
HL-IR	*Haemaphysalis longicornis* Ionotropic Receptor
GC-MS	Gas chromatography-Mass spectrometry
OBPs	Odorant-binding proteins
Orco	Odorant Receptor Co-receptor
ORx	Variable ligand-binding odorant receptor subunits
PMD	Para-menthane-3,8-diol
TRPA1	Transient Receptor Potential Ankyrin 1
SAR	Structure Activity Relationship

## References

[1] Dahiya N., Yadav M., Kataria D., Janjoter S., Sehrawat N. (2025). Mosquito-based transmission-blocking vaccine candidates for malaria: Progress, challenges, and innovations. Mol. Biol. Rep..

[2] Tang J., Amin M.A., Campian J.L. (2025). Past, present, and future of viral vector vaccine platforms: A comprehensive review. Vaccines.

[3] World Health Organization (2024). World Malaria Report 2024: Addressing Inequity in the Global Malaria Response. World Health Organization. https://www.who.int/publications/i/item/9789240104440.

[4] Diaz J.H. (2016). Chemical and plant-based insect repellents: Efficacy, safety, and toxicity. Wilderness Environ. Med..

[5] Fradin M.S., Day J.F. (2002). Comparative efficacy of insect repellents against mosquito bites. N. Engl. J. Med..

[6] Yadav D.K., Rathee S., Sharma V., Patil U.K. (2025). A comprehensive review on insect repellent agents: Medicinal plants and synthetic compounds. Anti-Inflamm. Anti-Allergy Agents Med. Chem..

[7] Hagstrum D.W., Phillips T.W., Cuperus G. (2012). Stored Product Protection (S156).

[8] Ghali H., Albers S.E. (2024). An updated review on the safety of N,N-diethyl-meta toluamide insect repellent use in children and the efficacy of natural alternatives. Pediatr. Dermatol..

[9] Lee M.Y. (2018). Essential oils as repellents against arthropods. BioMed Res. Int..

[10] Hazarika H., Krishnatreyya H. (2025). Technological advancements in mosquito repellents: Challenges and opportunities in plant-based repellents. Acta Parasitol..

[11] Tavares M., da Silva M.R.M., de Oliveira de Siqueira L.B., Rodrigues R.A.S., Bodjolle d’Almeida L., Dos Santos E.P., Ricci-Júnior E. (2018). Trends in insect repellent formulations: A review. Int. J. Pharm..

[12] Wu F., Chen Y., Gao M., Li W., Zhao Y., Wang Y. (2025). An updated review on essential oils from *Lauraceae* plants: Chemical composition and genetic characteristics of biosynthesis. Int. J. Mol. Sci..

[13] Shields V.D.C., Hildebrand J.G. (2001). Recent advances in insect olfaction, specifically regarding the morphology and sensory physiology of antennal sensilla of the female sphinx moth *Manduca sexta*. Microsc. Res. Tech..

[14] Heinbockel T.K., Alzyoud R.O., Raheel S., Shields V.D.C. (2026). Selected Essential Oils Act as Repellents Against the House Cricket, *Acheta domesticus*. Insects.

[15] Sritabutra D., Soonwera M. (2013). Repellent activity of herbal essential oils against *Aedes aegypti* (Linn.) and *Culex quinquefasciatus* (Say.). Asian Pac. J. Trop. Dis..

[16] Sathantriphop S., Kongmee M., Bangs M.J. (2015). The effects of plant essential oils on escape response and mortality rate of *Aedes aegypti* and *Anopheles minimus*. J. Vector Ecol..

[17] U.S. Environmental Protection Agency (n.d.). DEET (N,N-Diethyl-meta-toluamide). https://www.epa.gov/insect-repellents/deet.

[18] US Environmental Protection Agency (2014). DEET—Registration Review: Human Health and Environmental Risk Conclusions. [Docket EPA-HQ-OPP-2012-0162-0012]. https://www.epa.gov/insect-repellents/deet.

[19] Almeida A.R., Oliveira N.D., Pinheiro F.A.S.D., Morais W.A., Ferreira L.S. (2023). Challenges encountered by natural repellents: Since obtaining until the final product. Pestic. Biochem. Physiol..

[20] Calafat A.M., Baker S.E., Wong L.Y., Bishop A.M., Morales A.P., Valentin-Blasini L. (2016). Novel exposure biomarkers of N,N-diethyl-m-toluamide (DEET): Data from the 2007 2010 National Health and Nutrition Examination Survey. Environ. Int..

[21] Wickerham E.L., Lozoff B., Shao J., Kaciroti N., Xia Y., Meeker J.D. (2012). Reduced birth weight in relation to pesticide mixtures detected in cord blood of full-term infants. Environ. Int..

[22] Peng Y., Fang W., Krauss M., Brack W., Wang Z., Li F., Zhang X. (2018). Screening hundreds of emerging organic pollutants in surface water from the Yangtze River Delta: Occurrence, distribution, ecological risk. Environ. Pollut..

[23] Osimitz T.G., Murphy J.V. (1997). Neurological effects associated with use of the insect repellent N,N-diethyl-m-toluamide (DEET). J. Toxicol. Clin. Toxicol..

[24] Lipscomb J.W., Kramer J.E., Leikin J.B. (1992). Seizure following brief exposure to the insect repellent N,N-diethyl-m-toluamide. Ann. Emerg. Med..

[25] Shin N., Lascarez-Lagunas L.I., Henderson A.L., Martínez-García M., Karthikraj R., Barrera V., Sui S.H., Kannan K., Colaiácovo M.P. (2024). Altered gene expression linked to germline dysfunction following exposure of *Caenorhabditis elegans* to DEET. iScience.

[26] Yan S., Wang J., Xu J., Jiang W., Xiong M., Cao Z., Wang Y., Wang Z., Zhang T., Wang Z. (2022). Exposure to N,N-diethyl-m-toluamide and cardiovascular diseases in adults. Front. Public Health.

[27] Zhang H., Liu C., Sun Y., Tang S., Lei Y., Zhang W., Cheng B., Zhao Y., Luo Q. (2025). Toxicity assessment of N,N-diethyl-meta-toluamide (DEET) in zebrafish embryos. Comp. Biochem. Physiol. Part C Toxicol. Pharmacol..

[28] Cui Q., Zhu X., Guan G., Hui R., Zhu L., Wang J. (2022). Association of N,N-diethyl-m toluamide (DEET) with obesity among adult participants: Results from NHANES 2007–2016. Chemosphere.

[29] Wei C., He J., Wei Z., Huang Y., Xiong M., Deng C., Chen Z., Li W., Zhang X. (2023). Association between N,N-diethyl-m-toluamide exposure and the odds of kidney stones in U.S. adults: A population-based study. Front. Public Health.

[30] Wu R., Zhu X., Xing Y., Guan G., Zhang Y., Hui R., Cui Q., Liu Z., Zhu L. (2023). Association of N,N-diethyl-m-toluamide (DEET) with hyperuricemia among adult participants. Chemosphere.

[31] Liu L., Qin W., Nie L., Wang X., Dong X. (2024). Correlation between environmental DEET exposure and the mortality rate of cancer survivors: A large-sample cross-sectional investigation. BMC Cancer.

[32] US Department of Health and Human Services, Agency for Toxic Substances and Disease Registry (2017). Toxicological Profile for DEET (N,N-Diethyl-meta-toluamide). https://www.atsdr.cdc.gov/toxprofiles/tp185.pdf.

[33] Centers for Disease Control and Prevention (2024). Preventing Mosquito Bites. Mosquitoes.

[34] Strid A., Hanson W., Cross A., Bond C., Jenkins J. (2018). Insect Repellents Fact Sheet.

[35] Turek C., Stintzing F.C. (2013). Stability of Essential Oils: A Review. Compr. Rev. Food Sci. Food Saf..

[36] Yadav S.K. (2022). Physiochemical Properties of Essential Oils and Applications.

[37] Kessler A., Mueller M.B., Kalske A., Chautá A. (2023). Volatile-mediated plant–plant communication and higher-level ecological dynamics. Curr. Biol..

[38] Wu W., Yang Y., Feng Y., Ren X., Li Y., Li W., Huang J., Kong L., Chen X., Lin Z. (2022). Study of the repellent activity of 60 essential oils and their main constituents against *Aedes albopictus*, and nano-formulation development. Insects.

[39] Nerio L.S., Olivero-Verbel J., Stashenko E. (2010). Repellent activity of essential oils: A review. Bioresour. Technol..

[40] Bruce T.J., Pickett J.A. (2011). Perception of plant volatile blends by herbivorous insects: Finding the right mix. Phytochemistry.

[41] Uniyal A., Tikar S.N., Mendki M.J., Singh R., Shukla S.V., Agrawal O.P., Veer V., Sukumaran D. (2016). Behavioral response of *Aedes aegypti* mosquito towards essential oils using olfactometer. J. Arthropod-Borne Dis..

[42] Kamaraj C., Satish Kumar R.C., Al-Ghanim K.A., Nicoletti M., Sathiyamoorthy V., Sarvesh S., Ragavendran C., Govindarajan M. (2023). Novel essential oils blend as a repellent and toxic agent against disease-transmitting mosquitoes. Toxics.

[43] Ray A. (2015). Reception of odors and repellents in mosquitoes. Curr. Opin. Neurobiol..

[44] Webster B., Lacey E.S., Cardé R.T. (2015). Waiting with bated breath: Opportunistic orientation to human odor in the malaria mosquito, *Anopheles gambiae*, is modulated by minute changes in carbon dioxide concentration. J. Chem. Ecol..

[45] Paluch G., Bartholomay L., Coats J. (2010). Mosquito repellents: A review of chemical structure diversity and olfaction. Pest Manag. Sci..

[46] Meier C.J., Nguyen M.N., Potter C.J. (2025). Making scents of mosquito repellents. Trends Parasitol..

[47] Dhifi W., Bellili S., Jazi S., Bahloul N., Mnif W. (2016). Essential oils’ chemical characterization and investigation of some biological activities: A critical review. Medicines.

[48] Abbas M.G., Binyameen M., Azeem M., Majeed S., Sarwar Z.M., Nazir A., Sharif M.M.I., Parveen A., Mozūratis R. (2025). Chemical analysis, repellent, larvicidal, and oviposition deterrent activities of plant essential oils against *Aedes aegypti*, *Anopheles gambiae*, and *Culex quinquefasciatus*. Front. Insect Sci..

[49] Bohbot J.D., Dickens J.C. (2010). Insect repellents: Modulators of mosquito odorant receptor activity. PLoS ONE.

[50] Tsitoura P., Koussis K., Iatrou K. (2015). Inhibition of *Anopheles gambiae* odorant receptor function by mosquito repellents. J. Biol. Chem..

[51] Farina P., Conti B. (2024). Liabilities of essential oils as insect repellents. Curr. Opin. Environ. Sci. Health.

[52] Konopka J.K., Task D., Afify A., Raji J., Deibel K., Maguire S., Lawrence R., Potter C.J. (2021). Olfaction in *Anopheles* mosquitoes. Chem. Sens..

[53] Masyita A., Mustika Sari R., Dwi Astuti A., Yasir B., Rahma Rumata N., Emran T.B., Nainu F., Simal-Gandara J. (2022). Terpenes and terpenoids as main bioactive compounds of essential oils, their roles in human health and potential application as natural food preservatives. Food Chem. X.

[54] US Environmental Protection Agency (1997). R.E.D. Facts: Oil of Citronella (Reregistration Case 3105; PC Code 021901) (EPA-738-F-97-002).

[55] Katz T.M., Miller J.H., Hebert A.A. (2008). Insect repellents: Historical perspectives and new developments. J. Am. Acad. Dermatol..

[56] Corzo-Gómez J.C., Espinosa-Juárez J.V., Ovando-Zambrano J.C., Briones-Aranda A., Cruz Salomón A., Esquinca-Avilés H.A. (2024). A review of botanical extracts with repellent and insecticidal activity and their suitability for managing mosquito-borne disease risk in Mexico. Pathogens.

[57] Jaenson T.G.T., Garboui S., Pålsson K. (2006). Repellency of oils of lemon eucalyptus, geranium, and lavender and the mosquito repellent MyggA natural to *Ixodes ricinus* (Acari: Ixodidae) in the laboratory and field. J. Med. Entomol..

[58] Wiltz B.A., Suiter D.R., Gardner W.A. (2007). Deterrency and toxicity of essential oils to Argentine and red imported fire ants (Hymenoptera: Formicidae). J. Entomol. Sci..

[59] Buteler M., Alma A.M., Herrera M.L., Gorosito N.B., Fernández P.C. (2021). Novel organic repellent for leaf-cutting ants: Tea tree oil and its potential use as a management tool. Int. J. Pest Manag..

[60] Degenhardt J., Gershenzon J., Baldwin I.T., Kessler A. (2003). Attracting friends to feast on foes: Engineering terpene emission to make crop plants more attractive to herbivore enemies. Curr. Opin. Biotechnol..

[61] Gershenzon J., Dudareva N. (2007). The function of terpene natural products in the natural world. Nat. Chem. Biol..

[62] Sadgrove N.J., Padilla-González G.F., Phumthum M. (2022). Fundamental Chemistry of Essential Oils and Volatile Organic Compounds, Methods of Analysis and Authentication. Plants.

[63] Sharmeen J.B., Mahomoodally F.M., Zengin G., Maggi F. (2021). Essential Oils as Natural Sources of Fragrance Compounds for Cosmetics and Cosmeceuticals. Molecules.

[64] Ulland S., Borg-Karlson E.A.-K., Mustaparta H. (2006). Discrimination between enantiomers of linalool by olfactory receptor neurons in the cabbage moth *Mamestra brassicae* (L.). Chem. Senses.

[65] Hosseini M., Pereira D.M. (2023). The Chemical Space of Terpenes: Insights from Data Science and AI. Pharmaceuticals.

[66] Başer K.H.C., Buchbauer G. (2020). Handbook of Essential Oils: Science, Technology, and Applications.

[67] Benomari F.Z., Sarazin M., Chaib D., Pichette A., Boumghar H., Boumghar Y., Djabou N. (2023). Chemical Variability and Chemotype Concept of Essential Oils from Algerian Wild Plants. Molecules.

[68] Khan M.H., Dar N.A., Alie B.A., Dar S.A., Lone A.A., Mir G.H., Fayaz U., Ali S., Tyagi A., El-Sheikh M.A. (2023). Unraveling the Variability of Essential Oil Composition in Different Accessions of *Bunium persicum* Collected from Different Temperate Micro-Climates. Molecules.

[69] Reven M.E., Bowles E.J., Audia D.D., Cohen M.M., Joswiak D.J., Kurkas Lee B.A., May-Fitzgerald A.C., Peppers-Citizen M., Resnick J.A., Tomaino J.M. (2024). Quality Appraisal of Research Reporting for Aromatherapy and Essential Oil Studies in Humans: Proposed Checklist for “Transparent Reporting for Essential oil and Aroma Therapeutic Studies” (TREATS). J. Integr. Complement. Med..

[70] Sadgrove N., Jones G. (2015). A Contemporary Introduction to Essential Oils: Chemistry, Bioactivity and Prospects for Australian Agriculture. Agriculture.

[71] Zielińska-Błajet M., Feder-Kubis J. (2020). Monoterpenes and their derivatives- Recent development in biological and medical applications. Int. J. Mol. Sci..

[72] Bedini S., Flamini G., Ascrizzi R., Venturi F., Ferroni G., Bader A., Giraldi J., Conti B. (2018). Essential oils sensory quality and their bioactivity against the mosquito *Aedes albopictus*. Sci. Rep..

[73] Maia M.F., Moore S.J. (2011). Plant-based insect repellents: A review of their efficacy, development and testing. Malar. J..

[74] İşcan G. (2017). Antibacterial and anticandidal activities of common essential oil constituents. Rec. Nat. Prod..

[75] Müller G.C., Junnila A., Butler J., Kravchenko V.D., Revay E.E., Weiss R.W., Schlein Y. (2009). Efficacy of the botanical repellents geraniol, linalool, and citronella against mosquitoes. J. Vector Ecol..

[76] Carroll S.P., Loye J. (2006). PMD, a registered botanical mosquito repellent with deet-like efficacy. J. Am. Mosq. Control. Assoc..

[77] Melo N., Capek M., Arenas O.M., Afify A., Yilmaz A., Potter C.J., Laminette P.J., Para A., Gallio M., Stensmyr M.C. (2021). The irritant receptor TRPA1 mediates the mosquito repellent effect of catnip. Curr. Biol..

[78] Birkett M.A., Hassanali A., Hoglund S., Pettersson J., Pickett J.A. (2011). Repellent activity of catmint, *Nepeta cataria*, and iridoid nepetalactone isomers against Afro-tropical mosquitoes, ixodid ticks and red poultry mites. Phytochemistry.

[79] Reichert W., Ejercito J., Guda T., Dong X., Wu Q., Ray A., Simon J. (2019). Repellency assessment of *Nepeta cataria* essential oils and isolated nepetalactones on *Aedes aegypti*. Sci. Rep..

[80] Batume C., Mulongo I.M., Ludlow R., Ssebaale J., Randerso P., Pickett J.A., Mukisa I.M., Scofield S. (2024). Evaluating repellence properties of catnip essential oil against the mosquito species *Aedes aegypti* using a Y-tube olfactometer. Sci. Rep..

[81] Fichan I., Larroche C., Gros J.B. (1998). Water solubility, vapor pressure, and activity coefficients of terpenes and terpenoids. J. Chem. Eng. Data.

[82] Pub Chem National Library of Medicine, N.I.H.. https://pubchem.ncbi.nlm.nih.gov/.

[83] Keeling C., Bohlmann J. (2006). Diterpene resin acids in conifers. Phytochemistry.

[84] Sosa M.E., Tonn C.E., Giordano O.S. (1994). Insect antifeedant activity of clerodane diterpenoids. J. Nat. Prod..

[85] Li B.A., Li B.M., Bao Z., Li Q., Xing M., Li B. (2023). Dichlorodiphenyltrichloroethane for malaria and agricultural uses and its impacts on human health. Bull. Environ. Contam. Toxicol..

[86] Gebbinck E.A.K., Jansen B.J.M., de Groot A. (2002). Insect antifeedant activity of clerodane diterpenes and related model compounds. Phytochemistry.

[87] Li R., Morris-Natschke S.L., Lee K.H. (2016). Clerodane diterpenes: Sources, structures, and biological activities. Nat. Prod. Rep..

[88] Scheffrahn R.H., Hsu R.C., Su N.Y., Huffman J.B., Midland S.L., Sims J.J. (1988). Allelochemical resistance of bald cypress, *Taxodium distichum*, heartwood to the subterranean termite, *Coptotermes formosanus*. J. Chem. Ecol..

[89] Kusumoto N., Ashitani T., Hayasaka Y., Murayama T., Ogiyama K., Takahashi K. (2009). Antitermitic activities of abietane-type diterpenes from *Taxodium distichum* cones. J. Chem. Ecol..

[90] Nieto G., Ros G., Castillo J. (2018). Antioxidant and Antimicrobial Properties of Rosemary (*Rosmarinus officinalis,* L.): A Review. Medicines.

[91] McAndrew B.A. (1992). Sesquiterpenoids: The lost dimension of perfumery. Perfum. Flavorist.

[92] Barreto I.C., de Almeida A.S., Sena Filho J.G. (2021). Taxonomic Insights and Its Type Cyclization Correlation of Volatile Sesquiterpenes in Vitex Species and Potential Source Insecticidal Compounds: A Review. Molecules.

[93] Christensson J.B., Forsström P., Wennberg A.-M., Karlberg A.-T., Matura M. (2009). Air oxidation increases skin irritation from fragrance terpenes. Contact Dermat..

[94] Dittmar D., Schuttelaar M.L.A. (2019). Contact sensitization to hydroperoxides of limonene and linalool: Results of consecutive patch testing and clinical relevance. Contact Dermat..

[95] Clarkson T.C., Janich A.J., Sanchez-Vargas I., Markle E.D., Gray M., Foster J.R., Black W.C., Foy B.D., Olson K.E. (2021). Nootkatone Is an Effective Repellent against *Aedes aegypti* and *Aedes albopictus*. Insects.

[96] Gokulakrishnan J., Kuppusamy E., Shanmugam D., Appavu A., Kaliyamoorthi K. (2013). Pupicidal and repellent activities of *Pogostemon cablin* essential oil chemical compounds against medically important human vector mosquitoes. Asian Pac. J. Trop. Dis..

[97] da Silva R.C., Milet-Pinheiro P., Bezerra da Silva P.C., da Silva A.G., da Silva M.V., Navarro D.M., da Silva N.H. (2015). (E)-caryophyllene and α-humulene: *Aedes aegypti* oviposition deterrents elucidated by gas chromatography-electrophysiological assay of *Commiphora leptophloeos* leaf oil. PLoS ONE.

[98] Liakakou A., Angelis A., Papachristos D.P., Fokialakis N., Michaelakis A., Skaltsounis L.A. (2021). Isolation of volatile compounds with repellent properties against *Aedes albopictus* (Diptera: Culicidae) Using CPC Technology. Molecules.

[99] Kunert G., Reinhold C., Gershenzon J. (2010). Constitutive emission of the aphid alarm pheromone, (E)-β-farnesene, from plants does not serve as a direct defense against aphids. BMC Ecol..

[100] Rasmann S., Köllner T.G., Degenhardt J., Hiltpold I., Toepfer S., Kuhlmann U., Gershenzon J., Turlings T.C. (2005). Recruitment of entomopathogenic nematodes by insect-damaged maize roots. Nature.

[101] Noel J.P., Austin M.B., Bomati E.K. (2005). Structure-function relationships in plant phenylpropanoid biosynthesis. Curr. Opin. Plant Biol..

[102] Jankowska M., Rogalska J., Wyszkowska J., Stankiewicz M. (2017). Molecular Targets for Components of Essential Oils in the Insect Nervous System—A Review. Molecules.

[103] de Souza R.F., Amaro T.R., Palacio-Cortés A.M., da Silva M.A.N., Dionisio J.F., Pezenti L.F., Lopes T.B.F., Mantovani M.S., Zequi J.A.C., da Rosa R. (2024). Comparative transcriptional analysis between susceptible and resistant populations of *Aedes* (Stegomyia) *aegypti* (Linnaeus, 1762) after malathion exposure. Mol. Genet. Genom..

[104] Fabbro S.D., Nazzi F. (2013). From Chemistry to Behavior. Molecular Structure and Bioactivity of Repellents against *Ixodes ricinus* Ticks. PLoS ONE.

[105] Barrozo M.M., Santos E.F., Chagas H.D.F., Carvalho R.A., Silva I.S., de Souza Oliveira A., Faria L.C.F., Teixeira A.L.C., Zeringota V., Luz H.R. (2025). Repellent Activity of the Botanical Compounds Thymol, Carvacrol, Nootkatone, and Eugenol Against *Amblyomma sculptum* Nymphs. Pathogens.

[106] Kuang C., Cao J., Zhou Y., Zhang H., Wang Y., Zhou J. (2025). HL-TRP channel is required for various repellents for the parthenogenetic *Haemaphysalis longicornis*. Parasit. Vectors.

[107] Badji C.A., Dorland J., Kheloul L., Bréard D., Richomme P., Kellouche A., Azevedo de Souza C.R., Bezerra A.L., Anton S. (2021). Behavioral and Antennal Responses of *Tribolium confusum* to *Varronia globosa* Essential Oil and Its Main Constituents: Perspective for Their Use as Repellent. Molecules.

[108] Muhlemann J.K., Klempien A., Dudareva N. (2014). Floral volatiles: From biosynthesis to function. Plant Cell Environ..

[109] Lv M., Zhang L., Wang Y., Ma L., Yang Y., Zhou X., Wang L., Yu X., Li S. (2024). Floral volatile benzenoids/phenylpropanoids: Biosynthetic pathway, regulation and ecological value. Hortic. Res..

[110] Groux R., Hilfiker O., Gouhier-Darimont C., Peñaflor M.F., Erb M., Reymond P. (2014). Role of methyl salicylate on oviposition deterrence in *Arabidopsis thaliana*. J. Chem. Ecol..

[111] Gale C.C., Ferguson B., Rodriguez-Saona C., Shields V.D.C., Zhang A. (2024). Evaluation of a push-pull strategy for spotted-wing *Drosophila* management in highbush blueberry. Insects.

[112] Stevens G.R., Clark L. (1998). Bird repellents: Development of avian-specific tear gases for resolution of human–wildlife conflicts. Int. Biodeterior. Biodegrad..

[113] Hadani A., Ziv M., Rechav Y. (1977). A laboratory study of tick repellents. Entomol. Exp. et Appl..

[114] Hayden M.L., Rose G., Diduch K.B., Domson P., Chapman M.D., Heymann P.W., Platts-Mills T.A. (1992). Benzyl benzoate moist powder: Investigation of acaricidal [correction of acarical] activity in cultures and reduction of dust mite allergens in carpets. J. Allergy Clin. Immunol..

[115] Siddique A., Naeem J., Ang K.L., Abid S., Xu Z., Khawar M.T., Saleemi S., Abdullah M., Adeel (2024). Cinnamon and *Eucalyptus* Extracts: A Promising Natural Approach for Durable Mosquito-Repellent Fabrics with Multifunctionality. ACS Omega.

[116] Lopez A.D., Whyms S., Luker H.A., Galvan C.J., Holguin F.O., Hansen I.A. (2025). Repellency of essential oils and plant-derived compounds against *Aedes aegypti* mosquitoes. Insects.

[117] Popescu I.E., Gostin I.N., Blidar C.F. (2024). An Overview of the Mechanisms of Action and Administration Technologies of the Essential Oils Used as Green Insecticides. AgriEngineering.

[118] Barra A. (2009). Factors affecting chemical variability of essential oils: A review of recent developments. Nat. Prod. Commun..

[119] Yu W., Liu H., Liu M., Liu Z. (2000). Selective hydrogenation of citronellal to citronellol over polymer-stabilized noble metal colloids. React. Funct. Polym..

[120] Fitri N., Riza R., Akbari M.K., Khonitah N., Fahmi R.L., Fatimah I. (2022). Identification of citronella oil fractions as efficient bio-additive for diesel engine fuel. Designs.

[121] Krause S.T., Liao P., Crocoll C., Boachon B., Förster C., Leidecker F., Wiese N., Zhao D., Wood J.C., Buell C.R. (2021). The biosynthesis of thymol, carvacrol, and thymohydroquinone in Lamiaceae proceeds via cytochrome P450s and a short-chain dehydrogenase. Proc. Natl. Acad. Sci. USA.

[122] Hudaib M., Speroni E., Di Pietra A.M., Cavrini V. (2002). GC/MS evaluation of thyme (*Thymus vulgaris* L.) oil composition and variations during the vegetative cycle. J. Pharm. Biomed. Anal..

[123] Zinno P., Guantario B., Lombardi G., Ranaldi G., Finamore A., Allegra S., Mammano M.M., Fascella G., Raffo A., Roselli M. (2023). Chemical composition and biological activities of essential oils from *Origanum vulgare* genotypes belonging to the carvacrol and thymol chemotypes. Plants.

[124] Pluhár Z., Kun R., Cservenka J., Neumayer É., Tavaszi-Sárosi S., Radácsi P., Gosztola B. (2024). Variations in essential oil composition and chemotype patterns of wild thyme (*Thymus*) species in the natural habitats of Hungary. Horticulturae.

[125] Alfikri F.N., Pujiarti R., Wibisono M.G., Hardiyanto E.B. (2020). Yield, quality, and antioxidant activity of clove (*Syzygium aromaticum* L.) bud oil at the different phenological stages in young and mature trees. Scientifica.

[126] Behbahani B.A., Falah F., Arab F.A., Vasiee M., Tabatabaee F. (2020). Chemical composition and antioxidant, antimicrobial, and antiproliferative activities of *Cinnamomum zeylanicum* bark essential oil. Evid.-Based Complement. Altern. Med..

[127] Lee J., Choi D.B., Liu F., Grieco J.P., Achee N.L. (2018). Effect of the topical repellent para-Menthane-3,8-diol on blood feeding behavior and fecundity of the dengue virus vector *Aedes aegypti*. Insects.

[128] Solomon B., Sahle F.F., Gebre-Mariam T., Asres K., Neubert R.H.H. (2012). Microencapsulation of citronella oil for mosquito-repellent application: Formulation and in vitro permeation studies. Eur. J. Pharm. Biopharm..

[129] Higuchi C.T., Sales C.C., Andréo-Filho N., Martins T.S., Ferraz H.O., Santos Y.R., Lopes P.S., Grice J.E., Benson H.A.E., Leite-Silva V.R. (2023). Development of a Nanotechnology Matrix-Based Citronella Oil Insect Repellent to Obtain a Prolonged Effect and Evaluation of the Safety and Efficacy. Life.

[130] Sousa D.L., Xavier E.O., da Cruz R.C.D., de Souza I.A., de Oliveira R.A., da Silva D.C., Gualberto S.A., de Freitas J.S. (2023). Chemical composition and repellent potential of essential oil from *Croton tetradenius* (Euphorbiaceae) leaves against *Aedes aegypti* (Diptera: Culicidae). Biocatal. Agric. Biotechnol..

[131] Mukarram M., Choudhary S., Khan M.A., Poltronieri P., Khan M.M.A., Ali J., Kurjak D., Shahid M. (2022). Lemongrass Essential Oil Components with Antimicrobial and Anticancer Activities. Antioxidants.

[132] Dangol S., Poudel D.K., Ojha P.K., Maharjan S., Poudel A., Satyal R., Rokaya A., Timsina S., Dosoky N.S., Satyal P. (2023). Essential Oil Composition Analysis of *Cymbopogon* Species from Eastern Nepal by GC-MS and Chiral GC-MS, and Antimicrobial Activity of Some Major Compounds. Molecules.

[133] Haro-González J.N., Castillo-Herrera G.A., Martínez-Velázquez M., Espinosa-Andrews H. (2021). Clove Essential Oil (*Syzygium aromaticum* L. Myrtaceae): Extraction, Chemical Composition, Food Applications, and Essential Bioactivity for Human Health. Molecules.

[134] Silva M.V., de Lima A.D.C.A., Silva M.G., Caetano V.F., de Andrade M.F., da Silva R.G.C., de Moraes Filho L.E.P.T., de Lima Silva I.D., Vinhas G.M. (2024). Clove essential oil and eugenol: A review of their significance and uses. Food Biosci..

[135] Mitra S., Rodriguez S.D., Vulcan J., Cordova J., Chung H.-N., Moore E., Kandel Y., Hansen I.A. (2020). Efficacy of Active Ingredients From the EPA 25(B) List in Reducing Attraction of *Aedes aegypti* to Humans. J. Med. Entomol..

[136] Li Y.-Q., Kong D.-X., Wu H. (2013). Analysis and evaluation of essential oil components of cinnamon barks using GC–MS and FTIR spectroscopy. Ind. Crops Prod..

[137] Perdones A., Vargas M., Atarés L., Chiralt A. (2014). Physical, antioxidant and antimicrobial properties of chitosan–cinnamon leaf oil films as affected by oleic acid. Food Hydrocoll..

[138] Leyva-López N., Gutiérrez-Grijalva E.P., Vazquez-Olivo G., Heredia J.B. (2017). Essential Oils of Oregano: Biological Activity beyond Their Antimicrobial Properties. Molecules.

[139] Tabari M.A., Youssefi M.R., Maggi F., Benelli G. (2017). Toxic and repellent activity of selected monoterpenoids (thymol, carvacrol and linalool) against the castor bean tick, *Ixodes ricinus* (Acari: Ixodidae). Vet. Parasitol..

[140] Borugă O., Jianu C., Mişcă C., Goleţ I., Gruia A.T., Horhat F.G. (2014). *Thymus vulgaris* essential oil: Chemical composition and antimicrobial activity. J. Med. Life.

[141] Satyal P., Murray B.L., McFeeters R.L., Setzer W.N. (2016). Essential Oil Characterization of *Thymus vulgaris* from Various Geographical Locations. Foods.

[142] Najar B., Pistelli L., Ferri B., Angelini L.G., Tavarini S. (2021). Crop Yield and Essential Oil Composition of Two *Thymus vulgaris* Chemotypes along Three Years of Organic Cultivation in a Hilly Area of Central Italy. Molecules.

[143] Walasek-Janusz M., Grzegorczyk A., Malm A., Nurzyńska-Wierdak R., Zalewski D. (2024). Chemical Composition, and Antioxidant and Antimicrobial Activity of Oregano Essential Oil. Molecules.

[144] De Martino L., De Feo V., Formisano C., Mignola E., Senatore F. (2009). Chemical Composition and Antimicrobial Activity of the Essential Oils from Three Chemotypes of *Origanum vulgare* L. ssp. hirtum (Link) Ietswaart Growing Wild in Campania (Southern Italy). Molecules.

[145] Hudz N., Kobylinska L., Pokajewicz K., Horčinová Sedláčková V., Fedin R., Voloshyn M., Myskiv I., Brindza J., Wieczorek P.P., Lipok J. (2023). *Mentha piperita*: Essential Oil and Extracts, Their Biological Activities, and Perspectives on the Development of New Medicinal and Cosmetic Products. Molecules.

[146] Batish D.R., Singh H.P., Kohli R.K., Kaur S. (2008). Eucalyptus essential oil as a natural pesticide. For. Ecol. Manag..

[147] Barbosa L.C., Filomeno C.A., Teixeira R.R. (2016). Chemical variability and biological activities of *Eucalyptus* spp. essential oils. Molecules.

[148] Sheikh Z., Amani A., Basseri H.R., Kazemi S.H.M., Sedaghat M.M., Azam K., Azizi M., Amirmohammadi F. (2021). Repellent efficacy of *Eucalyptus globulus* and *Syzygium aromaticum*: Essential oils against malaria vector, *Anopheles stephensi* (Diptera: Culicidae). Iran. J. Public Health.

[149] Čmiková N., Galovičová L., Schwarzová M., Vukic M.D., Vukovic N.L., Kowalczewski P.Ł., Bakay L., Kluz M.I., Puchalski C., Kačániová M. (2023). Chemical Composition and Biological Activities of *Eucalyptus globulus* Essential Oil. Plants.

[150] Faria J.M.S., Pereira G., Figueiredo A.C., Barbosa P. (2025). In vivo and in vitro grown lemon-scented gum as a source of nematicidal essential oil compounds. Plants.

[151] Gillij Y.G., Gleiser R.M., Zygadlo J.A. (2008). Mosquito repellent activity of essential oils of aromatic plants growing in Argentina. Bioresour. Technol..

[152] Luker H.A., Salas K.R., Esmaeili D., Holguin F.O., Bendzus-Mendoza H., Hansen I.A. (2023). Repellent efficacy of 20 essential oils on *Aedes aegypti* mosquitoes and *Ixodes scapularis* ticks in contact-repellency assays. Sci. Rep..

[153] Oyarce G.A., Loyola P., Iubini-Aravena M., Romero Á., Rodríguez-Maciel J.C., Becerra J., Silva-Aguayo G. (2025). Adulticidal and repellent activity of essential oils from three cultivated aromatic plants against *Musca domestica* L.. Insects.

[154] Nenaah G.E., Alasmari S., Almadiy A.A., Albogami B.Z., Shawer D.A., Fadl A.E. (2023). Bio-efficacy of *Salvia officinalis* essential oil, nanoemulsion and monoterpene components as eco-friendly green insecticides for controlling the granary weevil. Ind. Crops Prod..

[155] Craft J.D., Satyal P., Setzer W.N. (2017). The chemotaxonomy of common sage (*Salvia officinalis*) based on the volatile constituents. Medicines.

[156] Mot M.-D., Gavrilaș S., Lupitu A.I., Moisa C., Chambre D., Tit D.M., Bogdan M.A., Bodescu A.-M., Copolovici L., Copolovici D.M. (2022). *Salvia officinalis* L. Essential oil: Characterization, antioxidant properties, and the effects of aromatherapy in adult patients. Antioxidants.

[157] Tabanca N., Wang M., Avonto C., Chittiboyina A.G., Parcher J.F., Carroll J.F., Kramer M., Khan I.A. (2013). Bioactivity-guided investigation of geranium essential oils as natural tick repellents. J. Agric. Food Chem..

[158] Rana V.S., Juyal J.P., Blázquez M.A. (2002). Chemical constituents of the essential oil of *Pelargonium graveolens* leaves. Int. J. Aromather..

[159] Sharopov F.S., Zhang H., Setzer W.N. (2014). Composition of geranium (*Pelargonium graveolens*) essential oil from Tajikistan. Am. J. Essent. Oils Nat. Prod..

[160] Yohana R., Chisulumi P.S., Kidima W., Tahghighi A., Maleki-Ravasan N., Kweka E.J. (2022). Anti-mosquito properties of *Pelargonium roseum* (Geraniaceae) and *Juniperus virginiana* (Cupressaceae) essential oils against dominant malaria vectors in Africa. Malar. J..

[161] Shellie R., Mondello L., Marriott P., Dugo G. (2002). Characterisation of lavender essential oils by using gas chromatography–mass spectrometry with correlation of linear retention indices and comparison with comprehensive two-dimensional gas chromatography. J. Chromatogr. A.

[162] Nedeltcheva-Antonova D., Gechovska K., Bozhanov S., Antonov L. (2022). Exploring the chemical composition of Bulgarian lavender absolute (*Lavandula Angustifolia* Mill.) by GC/MS and GC-FID. Plants.

[163] Pokajewicz K., Białoń M., Svydenko L., Fedin R., Hudz N. (2021). Chemical composition of the essential oil of the new cultivars of *Lavandula angustifolia* Mill. Bred in Ukraine. Molecules.

[164] Wińska K., Mączka W., Łyczko J., Grabarczyk M., Czubaszek A., Szumny A. (2019). Essential oils as antimicrobial agents—Myth or real alternative?. Molecules.

[165] Hammer K.A., Carson C.F., Riley T.V. (1999). Antimicrobial activity of essential oils and other plant extracts. J. Appl. Microbiol..

[166] Chouhan S., Sharma K., Guleria S. (2017). Antimicrobial activity of some essential oils—Present status and future perspectives. Medicines.

[167] Koyama S., Pham L., Murakawa Y., Ogawa Y., Terauchi K., Davis K. (2026). Unlocking the Power of Plant-Derived Natural Products: Therapeutic Benefits for Cognitive Health and Neuropsychiatric Symptoms in Dementia-Related Diseases. Plants.

[168] Donelli D., Antonelli M., Bellinazzi C., Gensini G.F., Firenzuoli F. (2019). Effects of lavender on anxiety: A systematic review and meta-analysis. Phytomedicine.

[169] Lee M.S., Choi J., Posadzki P., Ernst E. (2012). Aromatherapy for health care: An overview of systematic reviews. Maturitas.

[170] Tisserand R., Young R. (2014). Essential Oil Safety: A Guide for Health Care Professionals.

[171] Göbel H., Schmidt G., Soyka D. (1994). Effect of peppermint and eucalyptus oil preparations on neurophysiological and experimental algesimetric headache parameters. Cephalalgia.

[172] Bakkali F., Averbeck S., Averbeck D., Idaomar M. (2008). Biological effects of essential oils: A review. Food Chem. Toxicol..

[173] Mills S., Bone K. (2013). Principles and Practice of Phytotherapy: Modern Herbal Medicine.

